# Printing Accuracy and Flexural Properties of Different 3D-Printed Denture Base Resins

**DOI:** 10.3390/ma15072410

**Published:** 2022-03-24

**Authors:** Faisal D. al-Qarni, Mohammed M. Gad

**Affiliations:** Department of Substitutive Dental Sciences, College of Dentistry, Imam Abdulrahman Bin Faisal University, Dammam 31441, Saudi Arabia; mmjad@iau.edu.sa

**Keywords:** 3D printing, dimensional accuracy, flexural strength, dental prosthesis

## Abstract

Digital dentures can be fabricated by subtractive milling or, more recently, by 3D-printing technology. Several different 3D-printing technologies and materials are commercially available, and the differences in printing accuracy and mechanical behavior among them are unknown. Aim: This study evaluated the printing accuracy of 3D-printed denture base resins and assessed their flexural properties when compared with conventional heat-polymerized ones. Methods: A total of 40 acrylic specimens were prepared with four different materials: three 3D-printed resins, and a conventional heat polymerized resin was used as a control. The printing accuracy was evaluated by calculating the error rate of 3D-printed specimens compared with dimensions of the virtual design. Flexural strength and elastic modulus were assessed with a universal testing machine. One-way ANOVA and Kruskal–Wallis tests were used for analysis. Results: Printing accuracy across the tested materials was statistically different. Specimen length showed error rates between 1.3% and 2.4%, specimen width had error rates between 0.2% and 0.7%, and specimen thickness had error rates between 0.2% and 0.6%. Three-dimensional-printed specimens had lower flexural strength and elastic modulus values when compared with heat-polymerized specimens. Conclusions: The choice of material seems to influence printing accuracy, and to a lesser extent, flexural strength. However, it has no effect on the elastic modulus.

## 1. Introduction

Computer-aided design/computer-aided manufacturing (CAD/CAM) technology has recently been implemented for denture base production, through which dentures can be fabricated either by subtractive milling or by additive three-dimensional (3D) printing [1,2]. As the technology of 3D printing rapidly develops and has wider applications, more technological improvements can be anticipated. This would make the fabrication of well-fitting 3D-printed dentures an alternative to conventional and milling methods [3,4]. Additive 3D printing reported several advantages over subtractive milling, such as the ability to produce complex shapes, being more economical due to the lower material waste [5], and the fact that it does not demonstrate rotary burr wear [6,7].

Multiple factors reportedly influence the outcomes of 3D printing, which include the printing layer thickness, light source and intensity, as well as printing orientation. [5,7] Therefore, it is crucial to properly set printer parameters in order to produce optimal results [5,6]. It has previously been reported that the reproducibility and strength of 3D-printed prostheses depend upon the material type used for different prostheses fabrications [7]. Additionally, it was reported that the strength of the products varies with different materials under different conditions [8].

The satisfactory function of removable prostheses largely depends on the dimensional accuracy of the denture base resins, as it determines how well dentures fit over the underlying edentulous tissues, thus affecting prosthesis retention. Together with mechanical properties, dimensional accuracy could also influence the long-term denture success and frequency of required maintenance [9]. Although many previous investigations evaluated 3D-printed technology, data on the accuracy of 3D-printed denture base resin are still scarce. Shim et al. (2020) have investigated the accuracy of 3D-printed denture base resin with printing orientations as a variable. They stated that specimens printed at 90 degrees had the lowest error rates in length, while specimens printed at 45 degrees had the highest error rates in thickness [6]. In another study, the trueness of the 3D-printed denture base was reported to be superior to those made with subtractive milling [10].

Several laboratory-specific factors might affect the accuracy of 3D-printed resins, such as printing technologies, printing devices, and printing materials. [11] To attain optimal results, manufacturers specified certain printing parameters for their respective printers, and made recommendations for post-curing time and temperature for their post-curing devices [12,13]. Based on the findings of previous studies [12,13], this study hypothesized that there would be differences in the printing accuracy of different materials. Differences in the resolution of the printing axis are another possible reason for variations in the results. Consequently, the resolution in the different planes is characteristic for different 3D printers. The differences may also be due to the post-polymerization time, temperature, and curing machines used. These variations may be attributed to the differences in light intensity of the curing unit, as well as exposure time, since some curing units do not have a heating system [13].

Studies comparing the accuracy and strength of 3D-printed denture resins are sparse. Therefore, this study was conducted to evaluate the printing accuracy and flexural properties of different 3D-printed resins. The null hypothesis was that all tested 3D-printed materials would have similar printing accuracy, and their flexural properties would be comparable to that of conventional heat-polymerized materials.

## 2. Materials and Methods

### 2.1. Specimen Preparation

Specimens were prepared according to the International Organization for Standardization (ISO/FDIS 1567) [14,15] with 65 × 10 × 3.3 mm^3^ (±0.2 mm^3^) dimensions. Sample size was calculated using the World Health Organization’s formula, with level of significance set at 0.05 and 80% power, which revealed that 10 specimens/group would provide reliable evidence. A total of 40 specimens were prepared and divided into four main groups (n = 10): a control group (heat polymerized specimens), and 3 experimental 3D-printed resins groups:

1. DentaBASE (ASIGA, Erfurt, Germany);
2. Denture Base Resin LP (Formlabs Inc, Somerville, MA, USA);
3. Denture 3D+ (NextDent B.V., Soesterberg, The Netherlands).

Heat-polymerized acrylic specimens were prepared (Major.Base.20; Major Prodotti Dentari, Moncalieri, Italy) by investing wax specimens (with 65 × 10 × 3.3 mm^3^ dimension) in dental stone within flasks, followed by wax elimination, thus creating mold spaces. Acrylic resin powder and liquid were mixed, packed, and polymerized following manufacturer’s instructions, as described in a previous study [16]. After polymerization, specimens were finished and polished with 500- and 1200-grit silicon carbide grinding paper (Buehler, Lake Bluff, IL, USA). A digital caliper (Digital ABS AOS Caliper; Mitutoyo, Kawasaki, Japan) was used to verify the specimens’ dimensions.

A CAD virtual design was created with open-source software (123D design v. 2.2.14; Autodesk, CA, USA) following the same specimen dimensions (65 × 10 × 3.3 mm^3^ (±0.2 mm^3^)). The design was saved as a Standard Tessellation Language (STL) file and exported to 3D-printing software. Printing specifications, printers, and parameters are described in Table 1 and Table 2.

Once curing was completed, a slow-speed rotary instrument (Ti95L; NSK, Nakanishi inc., Kanuma, Japan) was used to carefully remove the specimens’ support structures, followed sequentially by standardized finishing with silicon carbide grinding papers (500-then 1200-grit) [17]. The finishing procedure was performed by one investigator. All specimens were immersed in distilled water for 48 (±2) h at 37 °C before testing [18].

### 2.2. Printing Accuracy

**Figure 1:**
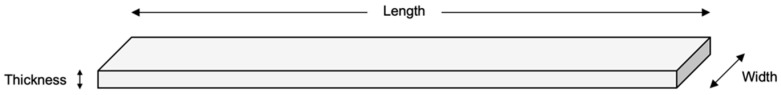
Illustrated specimen indicating the dimensions evaluated: length, width, and thickness.

A digital caliper (±0.1 mm) was used to measure length, width, and thickness for all specimens (Figure 1). All measurements were performed by one investigator. Each measurement was performed three times, and the average value was used for analysis. An average error percentage was calculated by comparing obtained measurements with those in the virtual CAD software [6].

### 2.3. Flexural Strength and Elastic Modulus

After accuracy measurements, the flexural strength and elastic modulus were evaluated using a three-point bending test following ISO standards, where specimens were subjected to compressive loading till fracture at a crosshead speed of 5.0 mm/min, using a Universal Testing Machine (ElectroPlusTM E3000; Instron, Buckinghamshire, UK). Flexural strength was calculated using the following formula [19]:

Flexural strength = 3FL/2bh^2^ (1)

where (F) is fracture load (N), (L) is the distance between the two supports, (b) is specimen width, and (h) is specimen thickness. Elastic modulus was calculated using the following formula [19]:

elastic modulus = FL^3^/4bh^3^d (2)

where the variables consist of the following: (F) is load (N), (L) is the distance between the two supports, (b) is specimen width, (h) is specimen thickness, and (d) is deflection in that point (p).

### 2.4. Statistical Analysis

Descriptive data were calculated as means and standard deviations. Normal distribution of data was verified with Shapiro–Wilk test. One-way ANOVA with Tukey’s post hoc test was used to evaluate the effect of materials used on printing accuracy (thickness and width), as well as flexural strength and elastic modulus. Kruskal–Wallis test was used to evaluate differences in printing accuracy (length) as data could not be assumed to be normally distributed. Significance level was set at *p* = 0.05, and calculations were made under the assumption that observations are independent. All analyses were completed with Statistical Package for the Social Sciences (SPSS v.26; IBM, Armonk, NY, USA).

## 3. Results

### 3.1. Printing Accuracy

**Figure 2:**
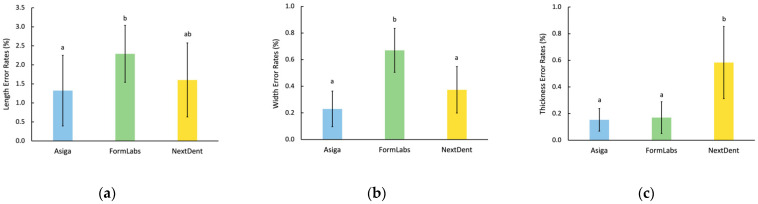
Printing accuracy of 3D-printed resin specimens. (**a**) Length; (**b**) Width; (**c**) Thickness. Similar letters indicate insignificant differences.

Specimen length had the highest error rate, followed by width and thickness. Differences in length dimensions among all groups were statistically significant (*p* = 0.035). Formlabs specimens had higher error rates in length than ASIGA specimens (*p* = 0.017), while NextDent specimens had no significant differences in comparison with ASIGA (*p* = 0.364), nor with Formlabs (*p* = 0.064). Specimen width measurements showed significant differences (*p* < 0.001), with Formlabs having higher error rates than ASIGA and NextDent (*p* < 0.001). However, ASIGA and NextDent specimens had similar error rates (*p* = 0.12). When specimen thickness was evaluated, the highest error rate was observed with NextDent (*p* < 0.001), while Formlabs and ASIGA were not statistically different (*p* = 0.97). Data are illustrated in Figure 2.

### 3.2. Flexural Strength and Elastic Modulus

**Figure 3:**
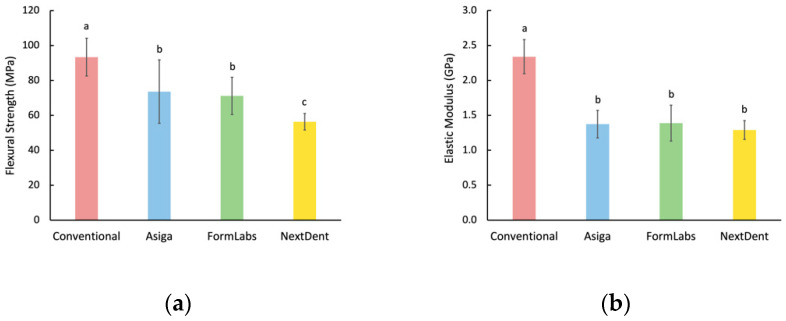
Flexural properties. (**a**) Flexural strength; (**b**) Elastic modulus. Similar letters indicate insignificant differences.

One-way ANOVA showed that flexural strength values had statistically significant differences among all groups (*p* < 0.001), with heat-polymerized specimens having the highest value (93.4 ± 10.8 MPa), while NextDent specimens showed the lowest flexural strength value (56.4 ± 4.7 MPa). Formlabs and ASIGA specimens had no significant differences between them (*p* = 0.458). The modulus of elasticity values had significant differences among tested groups (*p* < 0.001). Similar to flexural strength results, heat-polymerized specimens had a higher modulus of elasticity value than all other groups, with no statistical significance among them. Detailed flexural strength and elastic modulus data are illustrated in Figure 3.

Heat-polymerized specimens had the highest flexural strength value (93.4 ± 10.8 MPa), while NextDent specimens showed the lowest flexural strength value (56.4 ± 4.7 MPa). Formlabs and ASIGA specimens had no significant difference between them (*p* = 0.458). The modulus of elasticity values had significant differences among tested groups (*p* < 0.001). Similar to flexural strength results, heat-polymerized specimens had a higher modulus of elasticity value than all other groups, with no statistical significance among the experimental groups. Detailed flexural strength and elastic modulus data are illustrated in Figure 3.

## 4. Discussion

This study showed that printing accuracy varies based on the material selected. Additionally, flexural strength and elastic modulus values were different in comparison with heat-polymerized specimens. Therefore, the null hypothesis is rejected.

The use of digital technology to fabricate dental prostheses has been more popular in the past decade. Dentures that are conventionally fabricated by heat, microwave, and light polymerization can now also be milled or 3D-printed from CAD designs. Several studies have reported superior mechanical and surface properties of milled dentures compared with 3D-printed specimens. However, the nature of 3D printing is more precise and versatile, as well as being more economical, with less material waste. Several systems offer the fabrication of dentures through 3D printing, with the most reported ones being NextDent [1,20,21], Formlabs [22,23], and ASIGA [24], each with different printing technology. Little to no information exists as to whether the printing technology will affect various properties of the printed resin.

In terms of accuracy, this study showed that the length of the specimens was the most affected by the printing system, followed by width and thickness. This may be attributed to the printing orientation used, as printing was completed along specimens’ length. This may have resulted in more layer build up. A previous study reported that printing accuracy was directly influenced by the printing orientation used [7]. In addition, increased bonding and slight polymerization shrinkage, due to post-curing for further polymerization, may explain the high variations in the specimens’ length measurements. A previous study evaluated 3D-printed provisional crown and bridge dental materials with one printing system and three different printing orientations. It was shown that the highest error rate was with specimen width, followed by thickness, while specimen length was least affected [7]. More recently [6], another study evaluated different orientations of 3D-printed denture base materials and found that the highest error rate was associated with specimen thickness, followed by specimen width, then length. The results as to which dimension is more sensitive when fabricating 3D-printed specimens are conflicting and inconclusive. Further studies are needed to investigate the influence of different printing variables on printed object accuracy.

When comparing the 3D-printing systems that were investigated, Formlabs specimens had a higher error rate in width than NexDent, while in thickness, NexDent had a higher error rate than Formlabs. They were, however, similar in length error rates. ASIGA specimens, on the other hand, achieved the lowest error rates in all three dimensions. These differences could be explained by either the use of different liquid resins or different printing technologies adopted by the 3D-printing systems tested in this study. Since each of the tested materials was printed with the associated 3D printer from the same manufacturer, it is unclear whether it is the materials or the printer that had more impact. A study assessing different materials fabricated and using different printers is currently underway. A previous study evaluated the accuracy and trueness of dental models printed with different printing technologies: Stereolithography (SLA), digital light processing (DLP), and MultiJet printing (MJ). It reported similar precision for all three technologies; however, trueness was highest for models printed with NextDent [25]. In contrast, NextDent specimens in the present study had higher error rates than those made with DLP by ASIGA. The results, however, cannot be directly compared due to the different groups and assessment methods.

The accuracy of removable dentures is essential for the success of treatment, as retentive forces are largely dependent on intimate contact between the denture base and the underlying tissues [9]. Therefore, several previous studies evaluated denture accuracy with various assessment methods by measuring the gap between the posterior palatal denture base and the underlying master cast or measuring impression material that is dispensed between the denture and master cast [26]. Other studies evaluated linear shrinkage by measuring molar-to-molar distance [27]. The introduction of CAD/CAM materials through virtual CAD designs permitted the use of modern methods to measure accuracy through the software-based superimposition of fabricated specimens with CAD design [28]. With recent evolutions in CAD/CAM devices used in the dental field, it is essential to investigate different factors involved in the CAD/CAM process on the properties of fabricated prostheses. This study investigated common materials and printing technologies used for the fabrication of 3D-printed dentures and showed that there were significant influences on printing accuracy. However, the clinical significance of such results is yet to be determined, and further investigations on full-printed dentures with 3D analysis are needed.

The analysis of the flexural strength and elastic modulus values recorded in this study showed that heat-polymerized specimens performed better than those made with additive manufacturing. This is in agreement with other previous reports [1,21] and has been attributed to monomers in 3D-printed specimens. This also resulted in weaker double bonds [29], low adhesion between successive layers [30], and the formation of voids [31]. ASIGA and Formlabs specimens showed comparable flexural strength values, which were higher than NextDent specimens. These differing results may be attributed to different materials and/or different printing technologies used. In this study, NextDent specimens are the only ones that exhibited lower flexural strength than the ISO requirement of 65 MPa; however, the same material had higher values in other studies. An investigation of the chemical characterization of 3D-printed resins revealed that printing a certain material with the same printer could sometimes produce a different composition; thus, conflicting results of the same material can be expected [32]. The variability, repeatability, and longevity of flexural strength values of 3D-printed materials warrant further investigations.

The three common 3D-printed denture resins were investigated in the present study, which is considered a point of strength. However, accuracy measurements were performed for bar specimens, which may be different when whole dentures are tested. Another study limitation is the lack of thermal, water, or accelerated aging. Therefore, further investigations on full dentures fabricated with different resin materials, different printers, and different printing parameters are needed.

## 5. Conclusions

Within the limitations of this study, printing accuracy and flexural strength may be influenced by the material chosen, while the elastic modulus is less likely to be affected. Three-dimensional-printed specimens may exhibit lower flexural properties than those made with heat polymerization.

## Figures and Tables

**Table 1:** Three-dimensional-printed materials and printers used in this study.

Material	Printer	Manufacturer	Printing Technology
ASIGA DentaBASE	ASIGA MAX™	ASIGA, Erfurt, Germany	LED-based digital light processing (DLP)
Formlabs Denture Base Resin LP	Form 2	Formlabs Inc., Somerville, MA, USA	Stereolithography (SLA)
NextDent Denture 3D+	NextDent 5100	NextDent B.V., Soesterberg, The Netherlands	Figure 4 DLP

**Table 2:** Printing parameters used.

Group	Layer Thickness	Printing Orientation	Wavelength/Light Intensity	Post-Curing Rinse Solution	Post-Curing Machine	Post-Curing Time/Temperature
ASIGA	50 µm	90°	405 nm/ 13.14 mW/cm^2^	Isopropyl Alcohol 99.9%	LC-D Print Box (3D systems)	10 m/60 °C
Formlabs	50 µm	90°	395 nm/ 1.176 mW/cm^2^	Isopropyl Alcohol 99.9%	LC-D Print Box (3D systems)	10 m/60 °C
NextDent	50 µm	90°	405 nm/ 1.4 mW/cm^3^	Isopropyl Alcohol 99.9%	LC-D Print Box (3D systems)	10 m/60 °C

## Data Availability

Not applicable.
